# Koopman wavefunctions and classical–quantum correlation dynamics

**DOI:** 10.1098/rspa.2018.0879

**Published:** 2019-09-04

**Authors:** Denys I. Bondar, François Gay-Balmaz, Cesare Tronci

**Affiliations:** 1 Department of Physics and Engineering Physics, Tulane University, New Orleans, LA, USA; 2 CNRS and École Normale Supérieure, Laboratoire de Météorologie Dynamique, Paris, France; 3 Department of Mathematics, University of Surrey, Guildford, UK; 4 Mathematical Sciences Research Institute, Berkeley, CA, USA

**Keywords:** Koopman–von Neumann theory, quantum density matrix, classical–quantumdynamics

## Abstract

Upon revisiting the Hamiltonian structure of classical wavefunctions in Koopman–von Neumann theory, this paper addresses the long-standing problem of formulating a dynamical theory of classical–quantum coupling. The proposed model not only describes the influence of a classical system onto a quantum one, but also the reverse effect—the quantum backreaction. These interactions are described by a new Hamiltonian wave equation overcoming shortcomings of currently employed models. For example, the density matrix of the quantum subsystem is always positive definite. While the Liouville density of the classical subsystem is generally allowed to be unsigned, its sign is shown to be preserved in time for a specific infinite family of hybrid classical–quantum systems. The proposed description is illustrated and compared with previous theories using the exactly solvable model of a degenerate two-level quantum system coupled to a classical harmonic oscillator.

## 1. Introduction

Classical–quantum coupling has been an open problem since the rise of quantum mechanics. Bohr's concept of uncontrollable disturbance [1] affecting both classical and quantum systems during the measurement process has attracted much attention over the decades, and it would be unfeasible to provide here the enormous list of works in this field. The effect of the uncontrollable disturbance on the quantum system is often known under the name of ‘decoherence’ [2] and it manifests in terms of non-unitary dynamics and purity non-preservation [3]. Recently, the dynamics of a classical measuring device interacting with a quantum system has become a subject of experimental investigations (e.g. [4]). Over the last four decades, the apparent impossibility of a fully deterministic Hamiltonian description of classical–quantum coupling has been overcome by modelling decoherence in terms of Markov stochastic processes. Then, the quantum Lindblad equation [5,6] has emerged as the most general type of a Markovian master equation describing the evolution of a positive-definite and unit-trace quantum density matrix. Lindblad's theory, however, does not comprise the dynamics of the classical subsystem, which is simply treated as a thermodynamical bath.

In many physical contexts (e.g. in quantum chemistry and laser cooling), the systems under consideration are to be modelled as hybrid evolution to capture the coupling between electronic degrees of freedom and heavy nuclei. Then, it becomes essential to capture the ‘quantum backreaction’—the quantum feedback force on the evolution of the classical system (i.e. the nuclei). To this purpose, in 1981 Aleksandrov [7] and Gerasimenko [8] independently proposed the following quantum–classical Liouville equation for an operator-valued density on phase-space $\hat{D}(q,p,t)$:

1.1 $$\frac{\partial \hat{D}}{\partial t}=-i\hbar^{-1}[\hat{H},\hat{D}]+\frac{1}{2}\left(\{\hat{H},\hat{D}\}-\{\hat{D},\hat{H}\}\right),$$

where $\hat{H}(q,p)$ is the operator-valued Hamiltonian function and we have used the standard notation for commutators [ ,  ] and canonical Poisson brackets { ,  }. The work by Aleksandrov and Gerasimenko has been highly influential and its Wigner-transformed variant is currently used for modelling purposes [9]. Shortly after its appearance, the Aleksandrov–Gerasimenko (AG) equation (1.1) was rediscovered in [10], where it was derived from first principles in terms of invariance properties under canonical and unitary transformations. However, although equation (1.1) conserves the total energy $h=Tr\int \hat{H}\hat{D}\;d^{3}q\;d^{3}p$, the quantum density matrix $\int \hat{D}\;d^{3}q\;d^{3}p$ is not positive definite. More importantly, the AG equation lacks a Hamiltonian structure and this is due to the fact that the binary operation on the right-hand side of (1.1) does not satisfy the Jacobi identity, and thus it is not a type of Poisson bracket [11–13]. In this case, the absence of a Hamiltonian structure leads to time-irreversible dynamics [14], thereby indicating a possible entropy production, which should normally be formulated as an H-theorem. However, entropy-preserving dynamics requires the formulation of time-reversible models possessing a Hamiltonian structure, which is indeed available in the case of isolated classical and quantum systems. Then, a Hamiltonian model of quantum–classical hybrid dynamics becomes necessary to model recurrent evolution such as Rabi oscillations. Despite several efforts [15–23], Lie-algebraic arguments [11,13] tend to exclude the existence of a closed equation for $\hat{D}$ possessing a Hamiltonian structure (i.e. comprising the Jacobi identity).

Another stream of research on classical–quantum coupling goes back to Sudarshan's measurement theory [24] of 1976. Therein, Sudarshan proposed to couple classical and quantum dynamics by exploiting the Koopman–von Neumann (KvN) formulation of classical dynamics in terms of classical wavefunctions [25,26]. Rediscovered in several instances [27,28], this reformulation of classical mechanics has been attracting increasing attention [29–34] (Wilczek F 2015, unpublished data). See also [35] for a broad review of general applications of Koopman operators. In the KvN construction, the classical Liouville density *ρ*(**q**, **p**, *t*) is expressed as *ρ* = |*Ψ*|^2^, where *Ψ*(**q**, **p**, *t*) is a wavefunction obeying the KvN equation

1.2 $$i\hbar \frac{\partial \Psi }{\partial t}=\{i\hbar H,\Psi \}=:{\hat{L}}_{H}\Psi .$$

Here, we have introduced the Hermitian Liouvillian operator ${\hat{L}}_{H}\cdot =i\hbar \{H,\cdot \}$. A direct verification shows that the prescription *ρ* = |*Ψ*|^2^ returns the classical Liouville equation ∂_*t*_*ρ* = {*H*, *ρ*}. Upon working in the Heisenberg picture, Sudarshan extended equation (1.2) to include the interaction with quantum degrees of freedom by invoking special superselection rules to enforce physical consistency [24]. Although extremely inspiring, this approach has received some criticism over the years [36–39], mainly because the role of the superselection rules remains somewhat unclear. Still, one of the advantages of Sudarshan's proposal is that Koopman wavefunctions possess a simple canonical Hamiltonian structure formally equivalent to that underlying Schrödinger's equation. Indeed, this feature provides a great simplification over the AG approach, which instead is based on density operators and Wigner functions both carrying highly non-canonical Lie–Poisson brackets [40].

While several hybrid theories appearing in the literature may offer good approximations of classical–quantum coupling, a Hamiltonian theory is still lacking, and this poses specific problems concerning consistent transformation properties. This paper addresses the problem by following up on Sudarshan's idea of exploiting classical wavefunctions. Upon combining this approach with Hamiltonian methods, we shall show that KvN theory can be easily extended in such a way that its Hamiltonian functional coincides with the physical energy. In the second part of the paper, we shall infer a Hamiltonian theory for classical–quantum coupling by using the extended KvN representation within the context of geometric quantization. The proposed classical–quantum wave equation is illustrated on the exactly solvable model of a degenerate two-level quantum system quadratically coupled to a one-dimensional classical harmonic oscillator.

## 2. Koopman wavefunctions

We begin by looking at the Hamiltonian structure of the KvN equation (1.2). This structure is particularly transparent when looking at its variational formulation

2.1 $$\delta \int_{t_{1}}^{t_{2}}\int (\hbar \;Re(i\Psi^{*}\partial_{t}\Psi )-\Psi^{*}{\hat{L}}_{H}\Psi )\;d^{6}z\;dt=0,$$

which leads to a few observations.

First, the Hamiltonian functional for the KvN equation (1.2) is written as $h(\Psi )=\int \Psi^{*}{\hat{L}}_{H}\Psi \;d^{6}z=\hbar \int HIm\{\Psi^{*},\Psi \}\;d^{6}z$, where we have denoted ***z*** = (**q**, **p**). Then, we observe that the Hamiltonian functional for the KvN equation does not coincide with the total physical energy, which instead would read $\int H|\Psi {|}^{2}\;d^{6}z$ (according to the prescription *ρ* = |*Ψ*|^2^).

The second observation is that the quantity *Im*{Ψ*, *Ψ*} satisfies the classical Liouville equation and thus, in principle, we could set *ρ* = *Im*{Ψ*, *Ψ*}. Borrowing a terminology from fluid dynamics [41], this expression is often known as a *Clebsch representation* [42–44] in the context of geometric mechanics [45,46]. However, here we are left with the insurmountable problem that $\int Im\{\Psi^{*},\Psi \}\;d^{6}z=0$.

The third observation is more fundamental: we remark that the KvN Lagrangian (the integrand in (2.1)) is not covariant with respect to local phase transformations *Ψ*(***z***)↦e^i*φ*(***z***)^*Ψ*(***z***). However, this particular problem can be overcome by using the minimal coupling method in gauge theory. Let us introduce the multiplicative operator $\hat{Z}=z$ and its canonical conjugate $\hat{\Lambda }=-i\hbar \nabla$, and let us rewrite the Liouvillian as ${\hat{L}}_{H}=X_{H}(\hat{Z})\cdot \hat{\Lambda }$. Here, ***X***_*H*_ = *J*∇*H* is the classical Hamiltonian vector field and

$$J=\left(\begin{array}{cc} 0 & 1\\ -1 & 0 \end{array}\right),$$

so that $[{\hat{Z}}^{i},{\hat{\Lambda }}^{j}]=i\hbar \delta^{ij}$. Then, if (Φ,A) are the components of a *U*(1)-gauge potential, a gauge-covariant Liouvillian is constructed by the replacement

2.2 $$i\hbar \partial_{t}⟶i\hbar \partial_{t}-\Phi \;\mathrm{and}\;i\hbar \nabla ⟶i\hbar \nabla +A.$$

Then, the covariant Liouvillian takes the form

2.3 $${\hat{L}}_{H}:=\Phi (\hat{Z})+X_{H}(\hat{Z})\cdot (\hat{\Lambda }-A(\hat{Z})).$$

Now, the choice of gauge potential is usually prescribed in prequantization theory [47–49] as follows:

2.4 Φ(z)=H(z)andA(z)⋅dz=p⋅dq.

Here, the differential form A(z)⋅dz is known as the *symplectic potential*, so that the standard symplectic form is obtained as ω=−dA, or equivalently $\nabla A-{\left(\nabla A\right)}^{T}=-J$. Under the replacement (2.2), the variational principle (2.1) yields the modified KvN equation

2.5 $$i\hbar \frac{\partial \Psi }{\partial t}=\{i\hbar H,\Psi \}-(p\cdot \frac{\partial H}{\partial p}-H)\Psi .$$

First formulated in 1972 by Kostant [50], this equation has appeared in a few works [10,31,51,52], where it was noted that the expression *ρ* = |*Ψ*|^2^ again satisfies the classical Liouville equation. In addition, we emphasize that the phase term in equation (2.5) is readily seen to coincide with the Lagrangian

$$L=p\cdot \partial_{p}H-H,$$

thereby reminding us of the important relationship between phases and Lagrangians going back to Feynman's thesis [53]. The relationship between the Lagrangian and the classical phase is made explicit by replacing the polar form $\Psi =\sqrt{D}\;e^{iS/\hbar }$ in (2.5), thereby obtaining

$$\frac{\partial D}{\partial t}+\{D,H\}=0\;\mathrm{and}\;\frac{\partial S}{\partial t}+\{S,H\}=L.$$

Then, we recognize that, while KvN theory is totally equivalent to the classical Liouville equation, equation (2.5) also carries information about the classical phase. The crucial role of both classical and quantum phases was also exploited in connection to the Hamilton–Jacobi theory [54,55], although in that context the wavefunction is defined only on the position space.

As a further remark, we notice that different gauge fixings are possible as alternatives to (2.4). For example, the *harmonic oscillator gauge*

2.6 $$A\cdot dz=\frac{1}{2}Jz\cdot dz=\frac{1}{2}\left(p\cdot dq-q\cdot dp\right)$$

used in [47,52] is convenient for homogeneous quadratic Hamiltonians as in this case the corresponding phase term $\Phi -X_{H}\cdot A=H-z\cdot \nabla H/2$ vanishes identically. Moreover, since **p** · d**q** is also known as the ‘Liouville one-form’, we shall refer to the gauge in (2.4) as the *Liouville gauge*. Both gauges will be used in this paper, depending on convenience.

First appearing in van Hove's prequantization theory [49], the covariant Liouvillian ${\hat{L}}_{H}$ is known as a *prequantum operator* [56], and satisfies the Lie algebra relation $[{\hat{L}}_{H},{\hat{L}}_{K}]=i\hbar {\hat{L}}_{\left\{H,K\right\}}$. In addition, we have a one-to-one correspondence between the Hamiltonian *H* and the Hermitian operator ${\hat{L}}_{H}$ (unlike the correspondence $H\mapsto {\hat{L}}_{H}$, which is many-to-one). In the Heisenberg picture (here denoted by the superscript *H*), one defines ${\hat{L}}_{A}^{H}(t):=U{\left(t\right)}^{†}{\hat{L}}_{A}U(t)$, where $U(t)=\exp(-i{\hat{L}}_{H}t/\hbar )$ is the classical propagator for a given Hamiltonian *H*. Then, this yields $d{\hat{L}}_{A}^{\;H}/dt=i\hbar^{-1}[{\hat{L}}_{H},{\hat{L}}_{A}^{H}]$ as well as ${\hat{L}}_{H}^{H}={\hat{L}}_{H}$. By construction, one has the general property ${\hat{L}}_{A}^{H}={\hat{L}}_{A^{H}}$, where $A^{H}(t)=\exp(i{\hat{L}}_{H}t/\hbar )A$ and the Liouvillian ${\hat{L}}_{H}$ is given as in (1.2). See appendix A for further explanations. Therefore, the Heisenberg equation for ${\hat{L}}_{A}^{H}$ implies the usual dynamics d*A*^*H*^/d*t* = {*A*^*H*^, *H*} for classical observables.

Partly inspired by Kirillov [57], here we shall call (2.5) the *Koopman–van Hove (KvH) equation* and address the reader also to [31,52] for more discussions on how prequantization relates to KvN theory. Let us now examine the Hamiltonian structure of the modified KvN equation (2.5). The variational principle $\delta \int_{t_{1}}^{t_{2}}\int (\hbar \;Re(i\Psi^{*}\partial_{t}\Psi )-\Psi^{*}{\hat{L}}_{H}\Psi )\;d^{6}z\;dt=0$ determines the Hamiltonian functional

2.7 $$h=\int \Psi^{*}{\hat{L}}_{H}\Psi \;d^{6}z=\int H(|\Psi {|}^{2}+\mathrm{div}J)\;d^{6}z,$$

with

$$J=\Psi^{*}\;{\hat{Z}}_{+}\Psi \;\mathrm{and}\;{\hat{Z}}_{\pm }:=J(\pm \hat{\Lambda }-A).$$

We note in passing that the operators ${\hat{Z}}_{\pm }$ satisfy the commutation relations $[{\hat{Z}}_{\pm }^{i},\;{\hat{Z}}_{\pm }^{j}]=\mp i\hbar J^{ij}$ and $[{\hat{Z}}_{\pm }^{i},\;{\hat{Z\;}}_{\mp }^{j}]=0$, which were used in [29,58] (by adopting the harmonic oscillator gauge (2.6)) to rewrite quantum theory in terms of wavefunctions on phase-space. From equation (2.7), we see that the quantity $|\Psi {|}^{2}+divJ$ emerges as an alternative Clebsch representation for the Liouville density [59]. More specifically, this quantity is a momentum map [45,46,60,61] for the group of strict contact transformations generated by the operator $i\hbar^{-1}{\hat{L}}_{H}$ [49], where

2.8 $${\hat{L}}_{H}=H-\nabla H\cdot {\hat{Z}}_{+}.$$

While some of this material is illustrated in appendix A, we shall leave a more thorough discussion of these aspects for future work. Here, we emphasize that the momentum map property enforces the quantity $|\Psi {|}^{2}+divJ$ to satisfy the classical Liouville equation, as it can be verified by a direct and lengthy calculation.

At this point, given the expression of the total energy (2.7), we insist that this must be equal to the total physical energy $\int H\rho \;d^{6}z$, and thus we are led to the identification

2.9 $$\begin{array}{rl} \rho & =|\Psi {|}^{2}+div(\Psi^{*}{\hat{Z}}_{+}\Psi )\\ & =|\Psi {|}^{2}-div(JA\;|\Psi {|}^{2})+\hbar Im\{\Psi^{*},\Psi \}. \end{array}$$

Although we observe that this expression for the Liouville density is not positive definite, its sign is preserved in time since the Liouville equation is a characteristic equation. Remarkably, we notice that the term divJ does not contribute to the total probability, so that $\int \rho \;d^{6}z=\int |\Psi {|}^{2}\;d^{6}z=1$. On the other hand, the same divergence term does contribute to expectation values, so that, for example, $\langle z\rangle =\int z\rho \;d^{6}z=\int \Psi^{*}{\hat{Z}}_{-}\Psi \;d^{6}z$. As shown in [29], by adopting the harmonic oscillator gauge (2.6), this last relation returns the usual Ehrenfest equations for the expectation dynamics of canonical observables.

Lastly, we remark that the entire discussion can be repeated by replacing classical wavefunctions with (possibly unsigned) density-like operators mimicking von Neumann's density matrix [10]. Then, equation (2.5) is recovered upon setting $\hat{D}(z,z^{\prime },t)=\Psi (z,t)\Psi^{*}(z^{\prime },t)$ in the evolution equation $i\hbar \partial_{t}\hat{D}=[{\hat{L}}_{H},\hat{D}]$. In the following sections, we shall further extend the present gauge-covariant KvH construction to include the coupling to quantum degrees of freedom.

## 3. Hybrid classical–quantum dynamics

The formulation of hybrid classical–quantum dynamics is usually based on fully quantum treatments, in which some kind of factorization ansatz is invoked on the wave function. This ansatz is then followed by a classical limit on the factor that is meant to model the classical particle.

Here, we propose a different perspective: we shall start with the KvH construction for two classical particles and we shall perform a formal quantization procedure on one of them. This can be achieved in different ways, depending on the particular quantization procedure. For example, Gerasimenko proposed a similar approach in the context of Weyl quantization [8], while the KvH equation (2.5) was formulated by Kostant [50] in the context of geometric quantization [56,62]. Here, however, we shall adopt a simpler approach, which consists in a partial canonical quantization on the 2-particle Hamiltonian. We consider the KvH equation (2.5) for a wavefunction *Ψ*(***z***, ***ζ***) representing two particles with coordinates ***z*** = (**q**, **p**) and ***ζ*** = (***x***, ***μ***), and fix a Hamiltonian *H*(***z***, ***ζ***). Then, we apply canonical quantization only to the coordinates (***x***, ***μ***), so that one replaces $x\to \hat{x}$ (quantum position operator) and $\mu \to \hat{p}:=-i\hbar \partial_{x}$ (quantum momentum operator) in the 2-particle Hamiltonian *H*, which thus becomes an operator-valued function $\hat{H}(z,\hat{x},\hat{p})$ and the coordinate ***μ*** has been eliminated. The hybrid Hamiltonian is then replaced in (2.8) to obtain the hybrid Liouvillian ${\hat{L}}_{\hat{H}}=\hat{H}-\nabla \hat{H}\cdot \;{\hat{Z}}_{+}$, with ${\hat{Z}}_{+}=-J(i\hbar \nabla_{z}+A(z))$. Eventually, one is left with the following *classical–quantum wave equation* for the hybrid wavefunction (here, denoted by *Υ*(***z***, ***x***)):

3.1 $$i\hbar \partial_{t}\Upsilon =\hat{H}\Upsilon -\nabla \hat{H}\cdot \;{\hat{Z}}_{+}\Upsilon =:{\hat{L}}_{\hat{H}}\Upsilon .$$

For example, performing the partial quantization on the 2-particle Hamiltonian *H*(***z***, ***ζ***) = *p*^2^/2*M* + *μ*^2^/2*m* + *V* (**q**, ***x***) yields the hybrid classical–quantum Hamiltonian

3.2 $$\hat{H}=-\frac{\hbar^{2}}{2m}\Delta_{x}+\frac{1}{2M}p^{2}+V(q,x).$$

Equations with a similar structure to (3.1) were shown to occur in the Hamiltonian dynamics of quantum expectation values [63,64]. Equations similar to (3.1) were also obtained in [10] upon discarding the phase terms in the KvH equation (2.5), that is, by considering the standard KvN equation (1.2). In that paper the authors rejected their equations because of interpretative issues and the absence of a conserved positive energy. Here, we point out that, since ${\hat{L}}_{\hat{H}}$ is Hermitian, (3.1) is actually a Hamiltonian equation possessing a variational principle of the type

3.3 $$\delta \int_{t_{1}}^{t_{2}}Re\langle \Upsilon |(i\hbar \partial_{t}-{\hat{L}}_{\hat{H}})\Upsilon \rangle \;dt=0,$$

thereby preserving the energy invariant

3.4 $$h=\langle \Upsilon |{\hat{L}}_{\hat{H}}\Upsilon \rangle =Tr\int \Upsilon^{†}(z)\;{\hat{L}}_{\hat{H}}\Upsilon (z)\;d^{6}z.$$

Here, the dagger symbol denotes the adjoint in the quantum coordinates and similarly for the trace, so that $\langle \Upsilon_{1}|\Upsilon_{2}\rangle =Tr\int \Upsilon_{1}^{†}(z)\Upsilon_{2}(z)\;d^{6}z$.

Now we construct a generalized density operator $\hat{D}$ so that the total energy (3.4) reads $h=Tr\int \hat{H}\hat{D}\;d^{6}z$. Actually, the latter relation is obtained by a direct manipulation of the expression (3.4), upon defining

3.5 $$\begin{array}{rl} \hat{D}(z) & =\Upsilon (z)\Upsilon^{†}(z)+div(\Upsilon (z)\;{\hat{Z}}_{-}\Upsilon^{†}(z))\\ & =\Upsilon (z)\Upsilon^{†}(z)-\mathrm{div}(JA\Upsilon (z)\Upsilon^{†}(z))+i\hbar \{\Upsilon (z),\Upsilon^{†}(z)\}. \end{array}$$

This quantity plays the role of the AG generalized density in (1.1) and it belongs to the dual of the tensor product space of phase-space functions and Hermitian operators on the quantum state space. Since the latter tensor space is not a Lie algebra (notice $[{\hat{L}}_{\hat{F}},{\hat{L}}_{\hat{G}}]\neq {\hat{L}}_{\hat{K}}$ for some $\hat{K}(z)$), $\hat{D}$ does not carry a standard momentum map structure, and thus it cannot possess a closed Hamiltonian equation, in agreement with [11,13].

In addition, we remark that $\hat{D}$ is generally not positive definite and, unlike the purely classical case, its sign is not preserved in time. This feature (also occurring in the AG equation (1.1)) was justified in [10] by analogies with Wigner quasi-probability densities. In the present context, the quantum density matrix and the classical Liouville density read

3.6 $$\hat{\rho }=\int \hat{D}(z)\;d^{6}z=\int \Upsilon (z)\Upsilon^{†}(z)\;d^{6}z$$

and

3.7 $$\rho (z)=Tr\hat{D}(z)=Tr[\Upsilon (z)\Upsilon^{†}(z)+div(\Upsilon (z)\;{\hat{Z}}_{-\;}\Upsilon^{†}(z))].$$

Then, while the quantum density matrix is positive definite by construction (unlike the AG theory [7,8]), the classical Liouville density is allowed to become negative in the general case of classical–quantum interaction.

A further consequence of equation (3.1) is obtained by simply applying Ehrenfest's theorem; indeed, the latter yields the following expectation value equation for quantum–classical observables $\hat{A}(z)$:

3.8 $$i\hbar \frac{d\langle \hat{A}\rangle }{dt}=\langle \Upsilon \;|[{\hat{L}}_{\hat{A}},{\hat{L}}_{\hat{H}}]\Upsilon \rangle ,$$

where we have defined $\left\langle \hat{A}\rangle =Tr\int \hat{A}\hat{D}\;d^{6}z=\langle \Upsilon |{\hat{L}}_{\hat{A}}\Upsilon \right\rangle$. Then, the usual conservation laws are recovered in the case $[{\hat{L}}_{\hat{A}},{\hat{L}}_{\hat{H}}]=0$. For example, upon denoting $\hat{p}=-i\hbar \nabla_{x}$, the case $\hat{A}=p+\hat{p}$ recovers momentum conservation whenever the generic Hamiltonian (3.2) involves a translation-invariant potential $V(q-\hat{x})$. (Here, $\hat{x}$ denotes the quantum position operator.) Indeed, the conservation of total momentum $[{\hat{L}}_{p+\hat{p}},{\hat{L}}_{\hat{H}}]=0$ follows from the relations $[{\hat{L}}_{p},{\hat{L}}_{\hat{H}}]=i\hbar {\hat{L}}_{\left\{p,V\right\}}$ and $[{\hat{L}}_{\hat{p}},{\hat{L}}_{\hat{H}}]={\hat{L}}_{\left[\hat{p},V\right]}$, since we have $i\hbar \left\{p,V\right\}+[\hat{p},V]=0$. We remark that the expectation dynamics (3.8) differs from the corresponding result obtained from the AG equation (1.1).

We conclude by presenting the dynamics of $\hat{D}$. As we pointed out, $\hat{D}$ does not possess a closed Hamiltonian equation: this means that its evolution can only be expressed in terms of *Υ*. In the case of a finite-dimensional quantum state space, a lengthy computation shows that (in index notation)

3.9 $$\begin{array}{rl} \partial_{t}{\hat{D}}_{\alpha \beta } & =-i\hbar^{-1}[\hat{H},\hat{D}{]}_{\alpha \beta }+\{\hat{H},\hat{D}{\}}_{\alpha \beta }-\{\hat{D},\hat{H}{\}}_{\alpha \beta }\\ & \;+\{JA\Upsilon \Upsilon^{†},\nabla \hat{H}{\}}_{\alpha \beta }-\{\nabla \hat{H},JA\Upsilon \Upsilon^{†}{\}}_{\alpha \beta }\\ & \;+i\hbar^{-1}div[JA\cdot \nabla \hat{H},JA\Upsilon \Upsilon^{†}{]}_{\alpha \beta }+[JA\cdot \nabla \hat{H},\{\Upsilon ,\Upsilon^{†}\}{]}_{\alpha \beta }\\ & \;+div(\{{\hat{H}}_{\alpha \gamma },JA\Upsilon_{\beta }^{*}\}\Upsilon_{\gamma }-\{JA\Upsilon_{\alpha },{\hat{H}}_{\gamma \beta }\}\Upsilon_{\gamma }^{*})\\ & \;+\Upsilon_{\gamma }\{JA\cdot \nabla {\hat{H}}_{\alpha \gamma },\Upsilon_{\beta }^{*}\}-\{\Upsilon_{\alpha },JA\cdot \nabla {\hat{H}}_{\gamma_{\beta }}\}\Upsilon_{\gamma }^{*}\\ & \;-i\hbar \{\Upsilon_{\gamma },\{{\hat{H}}_{\alpha \gamma },\Upsilon_{\beta }^{*}\}\}+i\hbar \{\{\Upsilon_{\alpha },{\hat{H}}_{\gamma \beta }\},\Upsilon_{\gamma }^{*}\}, \end{array}$$

where all quantities are evaluated at ***z***. Despite the striking similarity between the first line above and the AG equation (1.1), the remaining terms in the $\hat{D}$-equation show that the classical–quantum interaction may be more involved than one might have expected. Nevertheless, the intricate nature of classical–quantum coupling becomes hidden by the formal simplicity of the following equations for the quantum and classical densities:

3.10 $$i\hbar \partial_{t}\hat{\rho }=\int [\hat{H},\hat{D}]\;d^{6}z\;\mathrm{and}\;\partial_{t}\rho =Tr\{\hat{H},\hat{D}\},$$

which coincide formally with the corresponding result obtained by using the AG equation (1.1). We notice, however, that the AG theory is fundamentally different from the classical–quantum model formulated here. As we already mentioned, the AG equation is not Hamiltonian and it does not generally preserve the positivity of the quantum density matrix $\hat{\rho }=\int \hat{D}(z)\;d^{6}z$. In addition, the classical–quantum wave equation (3.1) represents a significant simplification over the AG equation, since the solutions of (3.1) are defined on a lower dimensional space than the solutions of the AG equation.

## 4. Discussion

In this section, we discuss some of the consequences and implications of the classical–quantum wave equation (3.1). The first observation is about quantum decoherence, which naturally arises from the first in (3.10) in terms of purity non-preservation. Also, we observe that classical dynamics can be different from what we are used to in the absence of classical–quantum interaction. On the one hand, the last equation in (3.10) does not generally allow for point particle solutions. Since the latter are known to be classical pure states [23,65], we conclude that classical–quantum correlations induce a loss of classical purity that mimics quantum decoherence effects. This will be illustrated below on an exactly solvable example.

On the other hand, as we pointed out, the positivity of *ρ* may not be generally preserved in time [10]. Indeed, while the sign of *ρ* will be shown to be preserved for certain classes of hybrid Hamiltonians $\hat{H}$ (see §5), it is not possible to draw a similar conclusion in the general case. Although the emergence of a sign-indefinite *ρ* may seem surprising at first, an analogue of this situation can be readily found in the standard case of a harmonic oscillator interacting (by a linear or quadratic coupling) with a nonlinear quantum system. Let us consider the full quantum case in the Wigner representation: the Wigner–Moyal equation for *W*(***z***, ***ζ***) reads

$$\partial_{t}W={\left\{H,W\right\}}_{z}+{\left\{H,W\right\}}_{\zeta },$$

where { ,  } denotes the Moyal bracket in the set of coordinates given by the subscript. Here, *H*(***z***, ***ζ***) retains arbitrary nonlinear dependence on ***ζ***, while it is quadratic in ***z*** so that {*H*, *W*}_***z***_ = {*H*, *W*}_***z***_. We emphasize that, in the absence of the nonlinear quantum system, we have ∇_***ζ***_*H* = 0 and the oscillator undergoes classical evolution (while its quantum features are encoded in the initial condition). This means that the coupled system can be considered as equivalent to a hybrid classical–quantum system. Then, projecting out the quantum coordinates yields an equation for $\varrho (z)=\int W(z,\zeta )\;d^{6}\zeta$, that is, $\partial_{t}\varrho =\int \{H,W\}\;d^{6}\zeta$. This is exactly the analogue of our second equation in (3.10). Also in this case, despite the classical structure of the oscillator subsystem, its density ϱ may develop negative values in time (even if ϱ > 0 initially) because *W* is not generally positive. Then, as already pointed out by Feynman [66], the possibility of non-positive classical distributions in compound systems does not come as a surprise. Further discussions on the meaning of negative probabilities and their applications can be found, for example, in [66,67].

In addition, we wish to emphasize that, unlike Sudarshan's model [24], the present construction consistently recovers the mean-field model for the classical and quantum densities. This is readily verified by replacing the mean-field factorization ansatz *Υ*(***z***, ***x***) = *Ψ*(***z***)*ψ*(***x***) in the variational principle (3.3). Indeed, this operation returns

4.1 $$i\hbar \partial_{t}\Psi =\langle \psi |\hat{H}\psi \rangle \Psi -\nabla \langle \psi |\hat{H}\psi \rangle \cdot \;{\hat{Z}}_{+}\Psi$$

and

4.2 $$i\hbar \partial_{t}\psi =(\int \Psi^{*}{\hat{L}}_{\hat{H}}\Psi \;d^{6}z)\psi ,$$

so that the equations for the quantum density $\hat{\rho }=\psi \psi^{†}$ and the classical distribution *ρ* (as given in (2.9)) return the mean-field equations in the form

4.3 $$\partial_{t}\rho =\{Tr(\hat{\rho }\hat{H}),\rho \}\;\mathrm{and}\;i\hbar \partial_{t}\hat{\rho }=[\int \rho \hat{H}\;d^{6}z\;,\hat{\rho }].$$

We emphasize that here the mean-field model emerges as an exact closure obtained from the variational structure (3.3) of the classical–quantum wave equation (3.1). The same does not hold for the AG equation (1.1), which indeed lacks a variational formulation. As shown in [8], replacing the mean-field factorization ansatz $\hat{D}(z,t)=\hat{\rho }(t)\;\rho (z,t)$ in (1.1) yields an unclosed system, which then requires the extra closure condition of vanishing classical–quantum correlations.

Before concluding this section, it may be relevant to highlight that the whole construction presented here can also be reformulated in terms of a density-like operator. Indeed, one can simply replace the classical–quantum wave equation (3.1) by its correspondent for a positive-definite density-like operator $\hat{\Theta }$, that is,

$$i\hbar \partial_{t}\hat{\Theta }=[{\hat{L}}_{\hat{H}},\hat{\Theta }],$$

which we shall call the *classical–quantum von Neumann equation*. Given the level of difficulty of such an extension of the theory, in this paper we choose to leave this direction open for future work.

## 5. An exactly solvable system

Many studies use a linear classical–quantum interaction potential preventing quantum backreaction beyond mean-field effects. Indeed, in these cases, the force exerted on the classical degrees of freedom by the quantum subsystem does not depend on classical–quantum correlations. For example, in the case of the Jaynes–Cummings model, the expectation value dynamics for the classical momentum depends only on the spin expectation $\left\langle \hat{\sigma }\right\rangle$ (already occurring in the mean-field model), but not on mixed quantum–classical expectations, e.g. $\left\langle q\hat{\sigma }\right\rangle$. For the latter term to appear in the equation of the classical momentum expectation, a quadratic coupling between the classical and quantum subsystems is needed. Hence, to demonstrate the emergence of the quantum backreaction, we consider the exactly solvable case of a degenerate two-level quantum system quadratically coupled to a one-dimensional classical harmonic oscillator. The Hamiltonian of such a system reads

5.1 $$\hat{H}=H_{0}+\frac{q^{2}}{2}\alpha \cdot \hat{\sigma }\;\mathrm{and}\;H_{0}=\frac{p^{2}}{2m}+m\omega^{2}\frac{q^{2}}{2},$$

where *m* and *ω* denote, respectively, the mass and frequency of the harmonic oscillator, ${\hat{\sigma }}_{j}$ are the Pauli matrices (*j* = 1, 2, 3) representing the two-level quantum system and the vector ***α*** comprises the classical–quantum coupling constants *α*_*j*_. Since this example involves a harmonic oscillator, here we shall adopt the convenient gauge (2.6). In this case, the hybrid equation of motion (3.1) reads

5.2 $$\frac{\partial \Upsilon }{\partial t}=\left[q\left(m\omega^{2}+\alpha \cdot \hat{\sigma }\right)\frac{\partial }{\partial p}-\frac{p}{m}\frac{\partial }{\partial q}\right]\Upsilon ,$$

where $\Upsilon ={\left(\Upsilon_{1}(q,p,t),\Upsilon_{2}(q,p,t)\right)}^{T}\in C^{2}$. The equations for each component are decoupled after introducing the wavefunction $\tilde{\Upsilon }=\hat{U}\Upsilon$, where the unitary matrix $\hat{U}$ is defined by $\hat{U}\left(\alpha \cdot \hat{\sigma }\right){\hat{U}}^{†}=\lambda {\hat{\sigma }}_{3}$. In the last equation, we have used the fact that the matrix $\alpha \cdot \hat{\sigma }$ is traceless, thus its eigenvalues must be of equal magnitude but with the opposite sign. Then, solving each linear characteristic equation for each component ${\tilde{\Upsilon }}_{k}$ leads to the following exact solution of (5.2), expressed in terms of the initial condition *Υ*_0_ = *Υ*|_*t*=0_:

5.3 Υ=U^†(y1(ω+)y2(ω−)),

where $\omega_{\pm }=\sqrt{\omega^{2}\pm \lambda /m}$ and *y*_*l*_(*ω*_ ± _) denotes the component of the vector

5.4 $$y(\omega_{\pm })=\hat{U}\Upsilon_{0}\left(q=q\cos(\omega_{\pm }t)-\frac{p\sin(\omega_{\pm }t)}{m\omega_{\pm }},p=p\cos(\omega_{\pm }t)+m\omega_{\pm }q\sin(\omega_{\pm }t)\right).$$

**Figure 1.**
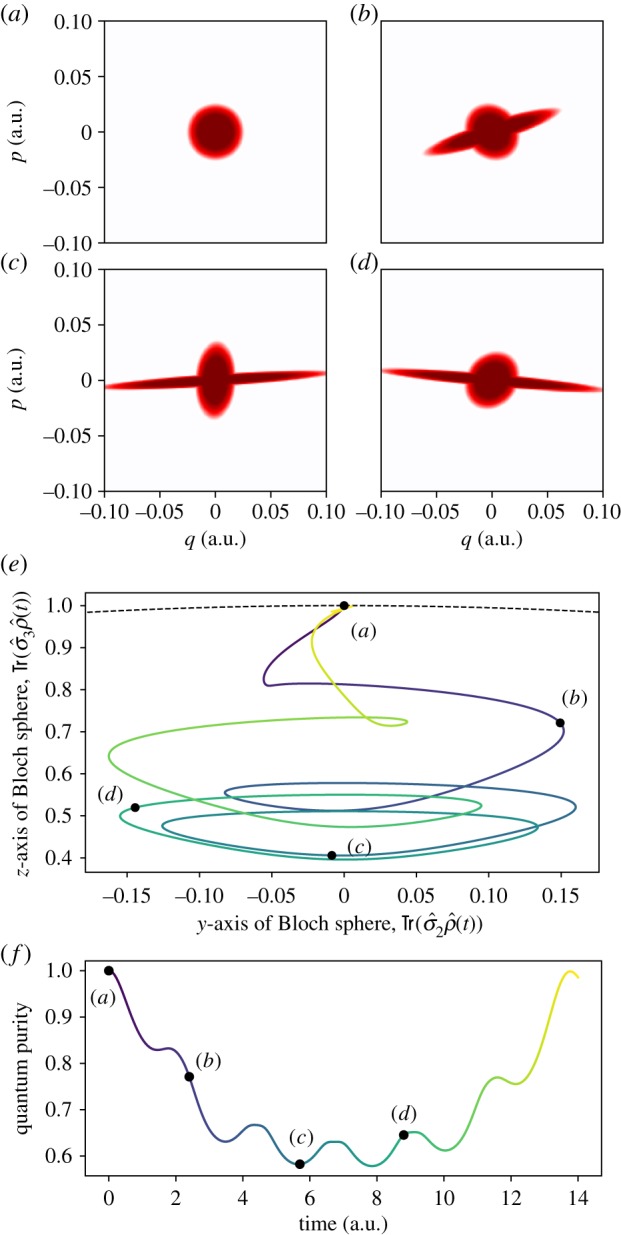
Hybrid evolution of a degenerate two-level quantum system quadratically coupled to a one-dimensional classical harmonic oscillator. The system Hamiltonian is given in (5.1). The depicted dynamics has the exact solution (5.3) with *ω* = *m* = 1 (a.u), ***α*** = (0.95, 0, 0) (a.u.), and the factorized initial condition (5.5) with *β* = 10^5^ (a.u.). The classical Liouville density (3.7) for this system is depicted at different times *t* = 0,  2.4,  5.7,  8.8 (a.u.) in the top panels (*a*), (*b*), (*c*) and (*d*), respectively. Red corresponds to positive values of the classical density $Tr\hat{D}$, whereas white marks vanishingly small values. (*e*) depicts the trajectory traced by the Bloch vector $n=Tr(\hat{\sigma }\hat{\rho })$ for the quantum density matrix (3.6) during the evolution. The progression of time is represented by a colour gradation from dark blue to bright yellow along the curve. Since the trajectory lies on the *yz* plane, only the *yz* projection is plotted. The dashed black line denotes the surface of the Bloch sphere. (*f* ) displays the purity $Tr({\hat{\rho }}^{2})=|n{|}^{2}$ of the quantum density matrix (3.6) as a function of time. In (*e*) and (*f* ), the captioned black dots mark time at which figures (*a*)–(*d*) are plotted. The colour encoding of time is the same in both (*e*) and (*f* ). (Online version in colour.)

Figure 1 depicts the classical–quantum evolution for such a system with the initial condition

5.5 Υ0=ω2π1−(1+βH0)e−βH0βH02 (10)andD^0=ωβ2πe−βH0(1  00  0),

corresponding to the uncorrelated quantum–classical state, where the quantum state (3.6) is the ground (i.e. ‘up’) state and the classical Liouville density (3.7) is the Boltzmann state *ρ*∝*e*^−*βH*_0_^ with an inverse thermodynamic temperature *β* and *H*_0_ as given in (5.1). The long-tailed wavefunction *Ψ* given by the square root in (5.5) and corresponding to the classical Boltzmann state can be easily obtained upon recalling (2.9) and by solving the differential equation $|\Psi {|}^{2}+div(\Psi^{*}{\hat{Z}}_{+}\Psi )=\omega \beta e^{-\beta H_{0}}/2\pi$. The latter is taken into a linear first-order ODE for |*Ψ*|^2^ by setting a zero phase and then changing to polar coordinates. We remark that the initial condition (5.5) represents a stationary state for the uncoupled classical–quantum system, that is, ***α*** = 0. See proposition 22.6 in [56] for the characterization of the stationary states of the KvH equation for the harmonic oscillator.

Figure 1 uses atomic units (a.u.), where the electron mass, the electron charge and ℏ are all set to unity.^1^ As can be seen, the quantum–classical correlations rapidly develop, yielding non-Gaussian classical Liouville densities (3.7) (due to the quantum backreaction) and non-pure quantum states (3.6). In other words, the classical system induces quantum decoherence (figure 1*f* ). It is noteworthy that the classical density is non-negative for all times in the considered example; we shall expand this particular point at the end of this section.

It is instructive to compare these findings with the predictions of the AG theory (1.1). The exact solution of the AG equation (1.1) for the Hamiltonian (5.1) reads in terms of the initial condition ${\hat{D}}_{0}=\hat{D}{|}_{t=0}$ as

5.6 $$\hat{D}={\hat{U}}^{†}\left(\begin{array}{cc} d_{11}(\omega_{+}) & e^{i\varphi }d_{12}(\omega )\\ e^{-i\varphi }d_{21}(\omega ) & d_{22}(\omega_{-}) \end{array}\right)\hat{U},$$

where *d*_*kl*_(*ω*_ ± _) denote the components of the matrix

5.7 $$\hat{d}(\omega_{\pm })=\hat{U}{\hat{D}}_{0}\left(q=q\cos(\omega_{\pm }t)-\frac{p\sin(\omega_{\pm }t)}{m\omega_{\pm }},p=p\cos(\omega_{\pm }t)+m\omega_{\pm }q\sin(\omega_{\pm }t)\right){\hat{U}}^{†}$$

and

5.8 $$\varphi =\frac{\lambda }{2m\hbar \omega^{3}}\left(\frac{p^{2}-{\left(m\omega q\right)}^{2}}{2m}\sin(2\omega t)-\omega \left[2H_{0}t+pq\left(\cos(2\omega t)-1\right)\right]\right).$$

**Figure 2.**
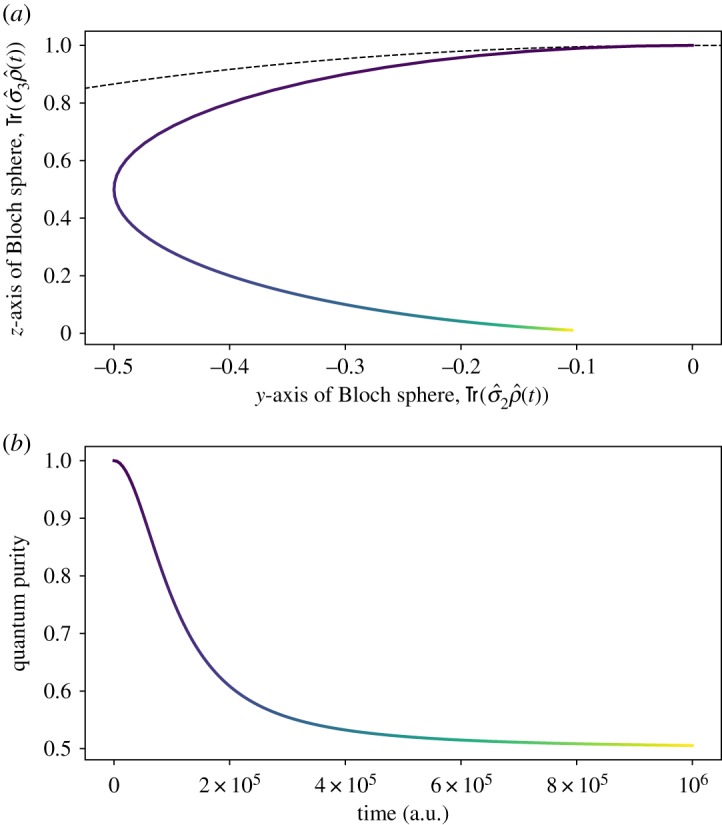
Hybrid evolution (5.6) governed by the AG equation (1.1) with the Hamiltonian given in (5.1) and the initial condition ${\hat{D}}_{0}$ in (5.5). The parameters used are the same as in figure 1. (*a*) depicts the trajectory traced by the Bloch vector for the quantum density matrix $\hat{\rho }=\int \hat{D}\;dpdq$ during the evolution. Similarly to figure 1, the progression of time is represented by a color gradation from dark blue to bright yellow. Again, the trajectory lies on the *yz* plane. However, we emphasize the very different time scale from the evolution displayed in figure 1. (*b*) displays the purity $Tr({\hat{\rho }}^{2})$ as a function of time. The color encoding of time is the same in both (*a*) and (*b*). The classical Liouville density $Tr\hat{D}$ is identical to figure 1*a*–*d*. (Online version in colour.)

The exact solutions (5.3) and (5.6) lead to qualitatively different dynamics. In particular, the phase *φ* breaks the time-reversible symmetry in AG hybrid dynamics. Furthermore, the term 2*H*_0_*t* in (5.8) yields a non-periodic evolution, which is responsible for the purity relaxation at large time scales, as shown in figure 2. The density matrix of the quantum subsystem monotonically approaches an infinite-temperature state. This dynamics is reminiscent of the relaxation predicted by the Lindblad equation modelling a dephasing channel. Indeed, in the case of the Lindblad equation, the entropy-driven relaxation process at macroscopic time scales (such as those in figure 2) is predicted by an H-theorem [68]. However, the lack of any features at microscopic time scales prevents the AG equation from capturing transient behaviour. This should be contrasted with the predictions of the new model depicted in figure 1, where recurrent quasi-periodic dynamics, akin to the Rabi oscillations, is observed with no long-time trend—a direct consequence of the model having the Hamiltonian structure. Despite these substantial differences, we emphasize that both the solutions (5.3) and (5.6) lead to the same classical Liouville density as shown in figure 1*a*–*d*. Another similarity between the two theories is that they both produce negative eigenvalues of the hybrid density $\hat{D}$. This fact was numerically verified for the considered example.

The parameters chosen in figures 1 and 2 are such that *β*≫2/(ℏ*ω*). This means that the initial condition (5.5) identifies a cold classical state, whose phase-space distribution in figure 1*a* violates the Heisenberg uncertainty principle. Therefore, figures 1 and 2 display truly hybrid dynamics, rendering quantum–classical correlations that cannot be modeled by the Pauli equation. However, if we set *β* = 2/(ℏ*ω*), the initial classical Liouville density *ρ* coincides with the Wigner function ${\left(\pi \hbar \right)}^{-1}\int \psi^{†}(q+s)\psi (q-s)\;e^{2ips/\hbar }\;ds$ for the Pauli spinor wavefunction *ψ*(*q*)∝*e*^−*mωq*^2^/(2ℏ) ^(1, 0)^*T*^. For such an initial condition, the classical density dynamics arising from equations of motion (1.1) and (5.2) coincides with the evolution of the Wigner function associated with the Pauli equation with Hamiltonian ${\hat{p}}^{2}/(2m)+m\omega^{2}{\hat{q}}^{2}/2+\alpha \cdot \hat{\sigma }{\hat{q}}^{2}/2$, where $[\hat{q},\hat{p}]=i\hbar$.

We conclude this section by showing that any hybrid Hamiltonian of the type $\hat{H}(q,p)=H_{0}(q,p)+V(q)\alpha \cdot \hat{\sigma }$ yields a hybrid wave equation (3.1) that preserves the sign of the classical Liouville density. By following the diagonalization procedure above, this class of hybrid Hamiltonians can be equivalently written as $\hat{H}=H_{0}+\lambda {\hat{\sigma }}_{3}V$, thereby producing two uncoupled KvH equations $i\hbar \partial_{t}{\tilde{\Upsilon }}_{\pm }={\hat{L}}_{H_{\pm }}{\tilde{\Upsilon }}_{\pm }$ of classical type (here, *H*_ ± _ = *H*_0_ ± *λV* ). From the arguments in §2, it follows that both these KvH equations preserve the sign of the quantity $\rho_{\pm }=|{\tilde{\Upsilon }}_{\pm }{|}^{2}+div({\tilde{\Upsilon }}_{\pm }^{*}{\hat{Z}}_{+}{\tilde{\Upsilon }}_{\pm })$. As a result, the sign of the classical density *ρ* = *ρ*_+_ + *ρ*_−_ of the hybrid system is also preserved in time. This result holds promise for other possible classes of hybrid Hamiltonians yielding positivity of the classical distribution; such a study is the subject of ongoing work [69].

## 6. Conclusion

Upon combining KvN classical mechanics with van Hove's prequantization theory, we have provided the new representation (2.9) of the Liouville density in terms of Koopman–van Hove classical wavefunctions. Then, given the KvH equation (2.5) for two particles, a quantization procedure was applied to one of them, thereby leading to the classical–quantum wave equation (3.1) for the hybrid wavefunction *Υ*(***z***, ***x***). This construction leads naturally to the identification of a sign-indefinite operator-valued density (3.5) encoding classical–quantum correlations. In turn, the latter can be discarded by invoking the factorization ansatz *Υ*(***z***, ***x***) = *Ψ*(***z***)*ψ*(***x***), recovering the celebrated mean-field model (4.3).

Equations (3.1), (3.5)–(3.7) constitute a long sought Hamiltonian model for classical–quantum hybrid evolution. As shown, the density matrix of the quantum subsystem is always positive, while the Liouville density of the classical subsystem may, in general, become negative in the general case. The proposed hybrid description has been illustrated and compared with the AG theory (1.1) by using the exactly solvable model of a degenerate two-level quantum system quadratically coupled to a one-dimensional classical harmonic oscillator. In this case, the quantum backreaction leads to positive-definite, yet non-Gaussian classical distributions. The discussion of which classes of hybrid systems preserve the sign of the classical distribution is left for future work [69]. Other questions currently under study [69] involve the algebraic structure of the hybrid correspondence $\hat{H}\to {\hat{L}}_{\hat{H}}$ and the associated dual map yielding the hybrid density $\hat{D}$.

As a further direction, we plan to develop effective numerical schemes for the classical–quantum wave equation (3.1) to be able to assess its physical consequences in experimentally relevant scenarios, such as those involving the Jaynes–Cummings model. In addition, the identification of hybrid classical–quantum thermal equilibria is an interesting question whose answer may open new perspectives in the statistical mechanics of hybrid classical quantum systems [70]. Indeed, once a Hamiltonian model is established, the immediate next question involves its extension to time-irreversible processes governed by an H-theorem. We remark that time irreversibility and energy dissipation are substantially different phenomena which may or may not coexist. Examples are given by the quantum Lindblad equation and the classical Botzmann equation, respectively. The addition of thermodynamic effects to Hamiltonian theories is a challenging question requiring methods from statistical mechanics. We leave this important direction for future work.

## Footnotes

1 All the source codes used to arrive at the results (including plotting) of this section can be found at https://github.com/dibondar/QCHybrid.

## Data accessibility

This article does not contain any additional data.

## Appendix A. KvH momentum map

In this section, we show explicitly that relation (2.9) identifies a momentum map for the infinitesimal action given by the operator ${\hat{L}}_{H}$. In geometric mechanics [45,46], momentum maps [60,61] represent a generalization of Noether's theorem to canonical group actions that are not necessarily a symmetry of the system under consideration. In this context, the Noether charge is generalized to a momentum map that evolves under the coadjoint representation associated with the Lie group acting on the considered mechanical system.

Without entering further details, we define the momentum map on symplectic vector spaces as follows. Let (*V*, *Ω*) be a vector space with constant symplectic form *Ω* and let the latter be preserved by a *G*-group representation on *V* . Then, the momentum map $J:V\mapsto g^{*}$ taking values in the dual space $g^{*}$ of the Lie algebra g of *G* is defined as

$$2\langle J(v),\xi \rangle :=\Omega (\xi_{V}(v),v),$$

where ξ∈g, *ξ*_*V*_ denotes the infinitesimal action on *V* , and 〈 · , · 〉 is the real-valued duality pairing for g. The momentum map **J**(*v*) is generally called a *Clebsch representation*.

In our case, *V* is the space of classical wavefunctions, the Lie algebra is the space $g=C^{\infty }(R^{6})$ of phase-space functions (endowed with the canonical bracket and the standard *L*^2^-pairing) and the infinitesimal generator *ξ*_*V*_(*v*) reads $-i\hbar^{-1}{\hat{L}}_{H}\Psi$. Then, upon using the Schrödinger (canonical) symplectic form $\Omega (\Psi_{1},\Psi_{2})=2\hbar Im\int \Psi_{1}^{*}(z)\Psi_{2}(z)\;d^{6}z$, the definition of the momentum map reads

$$\int HJ(\Psi )\;d^{6}z=\int \Psi^{*}{\hat{L}}_{H}\Psi \;d^{6}z.$$

Therefore, we compute

$$\begin{array}{rl} \int \Psi^{*}{\hat{L}}_{H}\Psi \;d^{6}z & =\int \Psi^{*}[\{i\hbar H,\Psi \}+(H-A\cdot J\nabla H)\Psi ]\;d^{6}z\\ & =\int [|\Psi {|}^{2}-div(JA|\Psi {|}^{2})+i\hbar \{\Psi ,\Psi^{*}\}]H\;d^{6}z. \end{array}$$

Now, we observe that $i\hbar \{\Psi ,\Psi^{*}\}=-i\hbar div(\Psi^{*}J\nabla \Psi )=div(\Psi^{*}J\hat{\Lambda }\Psi )$ so that the momentum map reads

$$J(\Psi )=|\Psi {|}^{2}+div[\Psi^{*}J(\hat{\Lambda }-A)\Psi ],$$

thereby recovering relation (2.9) as a Clebsch representation. By proceeding analogously, we notice that |*Ψ*|^2^ is also a Clebsch representation generated by local phase transformations with infinitesimal action *ξ*_*V*_(*v*) given as −iℏ^−1^*ϕΨ* (where *ϕ*(***z***) is a real phase-space function).

Notice that, since $-i\hbar^{-1}{\hat{L}}_{H}$ is skew-Hermitian, the correspondence $H\mapsto -i\hbar^{-1}{\hat{L}}_{H}$ provides a Lie algebra homomorphism between phase-space functions endowed with the canonical Poisson bracket and skew-Hermitian operators on classical wavefunctions. Then, the map −iℏ*Ψ*(***z***)*Ψ**(***z***′)↦**J**(*Ψ*) emerges as the dual of this Lie algebra homomorphism, thereby ensuring infinitesimal equivariance of ***J***(*Ψ*) and the consequent Poisson mapping property [45,46]. Thus, this guarantees that the momentum map **J**(*Ψ*) obeys the classical Liouville equation. Again, without entering further details, here we only mention that the operator $-i\hbar^{-1}{\hat{L}}_{H}$ emerges as the infinitesimal generator of a Lie group representation first discussed in van Hove's thesis [49], which is at the heart of classical mechanics. Under the name of ‘strict contact transformations’, this Lie group is a central extension of standard canonical transformations. This and related points will be discussed in more detail in future work.

An important consequence of the fact that the operator $-i\hbar^{-1}{\hat{L}}_{H}$ generates strict contact transformations (as opposed to $-i\hbar^{-1}{\hat{L}}_{H}$, which generates canonical transformations) is the equivariance property resulting as a general property of infinitesimal generators associated with group actions. As discussed in §2, in the Heisenberg picture we have ${\hat{L}}_{A}^{\;H}={\hat{L}}_{A^{H}}$, where ${\hat{L}}_{A}^{H}(t):=\exp(i{\hat{L}}_{H}t/\hbar ){\hat{L}}_{A}\exp(-i{\hat{L}}_{H}t/\hbar )$ and $A^{H}(t)=\exp(i{\hat{L}}_{H}t/\hbar )A$. Indeed, this is a consequence of the general formula (Ad_*g*_*ξ*)_*V*_ = *Φ**_*g*^−1^_*ξ*_*V*_ for a left representation *Φ* of a Lie group *G* on a vector space *V* . In the specific case under consideration, the adjoint action Ad_*g*_*ξ* coincides with the pushforward *A*°*η*^−1^ of the function *ξ* = *A* by the canonical transformation $\eta^{-1}=\exp(-X_{H}t)$ generated by $i\hbar^{-1}{\hat{L}}_{H}$, so that Ad_*η*_*A* = *A*°*η*^−1^. Therefore, we write $A\circ \eta^{-1}=\exp(i{\hat{L}}_{H}t/\hbar )A$.

## Appendix B. Hybrid dynamics

In this appendix, we provide calculational details of the discussion concerning classical–quantum hybrids. Here A is an arbitrary potential with dA=−ω or, equivalently, $\nabla A-{\left(\nabla A\right)}^{T}=-J$. First, we shall show that definition (3.5) leads to rewriting the total energy (3.4) as

$$h=Tr\int \Upsilon^{†}(z)\;{\hat{L}}_{\hat{H}}\Upsilon (z)\;d^{6}z=Tr\int \hat{H}\hat{D}\;d^{6}z,$$

with D given in (3.5). Indeed, we verify this as follows:

$$\begin{array}{rl} Tr\int \Upsilon^{†}\;[\hat{H}-\nabla \hat{H}\cdot J(\hat{\Lambda }-A)]\Upsilon \;d^{6}z & =Tr\int [\Upsilon \Upsilon^{†}\hat{H}-div(JA\Upsilon \Upsilon^{†})\hat{H}+i\hbar \Upsilon^{†}\{\hat{H},\Upsilon \}]d^{6}z\\ & =Tr\int [\Upsilon \Upsilon^{†}\hat{H}-div(JA\Upsilon \Upsilon^{†})\hat{H}-\hat{H}div(\Upsilon J\hat{\Lambda }\Upsilon^{†})]d^{6}z\\ & =Tr\int [\Upsilon \Upsilon^{†}+div(\Upsilon \;{\hat{Z}}_{-}\Upsilon^{†})]\hat{H}\;d^{6}z, \end{array}$$

where all quantities are evaluated at ***z*** and we used

$$i\hbar Tr\int \Upsilon^{†}\{\hat{H},\Upsilon \}\;d^{6}z=i\hbar Tr\int \hat{H}\{\Upsilon ,\Upsilon^{†}\}\;d^{6}z=Tr\int \hat{H}div(\Upsilon (i\hbar J\nabla )\Upsilon^{†})\;d^{6}z.$$

In conclusion, we recover definition (3.5).

Now we want to prove the $\hat{D}$-equation (3.9). For this purpose, we shall use the adjoint of equation (3.1), that is,

$$-i\hbar \partial_{t}\Upsilon^{†}(z)=\Upsilon^{†}(z)\hat{H}(z)-(\;{\hat{Z}}_{-}\Upsilon^{†}(z))\cdot \nabla \hat{H}(z),$$

which arises from the relation ${\left({\hat{Z}}_{+}\Upsilon (z)\right)}^{†}=\;{\hat{Z}}_{-}\Upsilon^{†}(z)$. At this point, we restrict ourselves to finite dimensions and, upon taking the time derivative of definition (3.5), one obtains

$$\begin{array}{rl} \partial_{t}\hat{D} & =\frac{d}{dt}\left(\Upsilon \Upsilon^{†}-div\left(JA\;\Upsilon \Upsilon^{†}\right)+i\hbar \{\Upsilon ,\Upsilon^{†}\}\right)\\ & =-i\hbar^{-1}(\hat{H}+JA\cdot \nabla \hat{H})\Upsilon \Upsilon^{†}+\{\hat{H},\Upsilon \}\Upsilon^{†}\\ & \;+i\hbar^{-1}\Upsilon \Upsilon^{†}(\hat{H}+JA\cdot \nabla \hat{H})-\Upsilon \{\Upsilon^{†},\hat{H}\}\\ & \;-div\left(JA\left(-i\hbar^{-1}(\hat{H}+JA\cdot \nabla \hat{H})\Upsilon \Upsilon^{†}+\{\hat{H},\Upsilon \}\Upsilon^{†}\right)\right)\\ & \;-div\left(JA\left(i\hbar^{-1}\Upsilon \Upsilon^{†}(\hat{H}+JA\cdot \nabla \hat{H})-\Upsilon \{\Upsilon^{†},\hat{H}\}\right)\right)\\ & \;+i\hbar \left\{\left(-i\hbar^{-1}(\hat{H}+JA\cdot \nabla \hat{H})\Upsilon +\{\hat{H},\Upsilon \}\right),\Upsilon^{†}\right\}\\ & \;+i\hbar \left\{\Upsilon ,\left(i\hbar^{-1}\Upsilon^{†}(\hat{H}+JA\cdot \nabla \hat{H})-\{\Upsilon^{†},\hat{H}\}\right)\right\}. \end{array}$$

We recall that in the present notation all quantities are evaluated at ***z***, e.g. *ΥΥ*^†^ stands for *Υ*(***z***)*Υ*^†^(***z***). We expand the divergence $div[\hat{H}+\nabla \hat{H}\cdot JA,\Upsilon \Upsilon^{†}JA]$ in the fourth and fifth lines and we use the Leibniz product rule and the Jacobi identity in the last two lines. Then, a few cancellations yield

$$\begin{array}{rl} \partial_{t}{\hat{D}}_{\alpha \beta } & =-i\hbar^{-1}[\hat{H},\Upsilon \Upsilon^{†}-div(JA\Upsilon \Upsilon^{†}){]}_{\alpha \beta }+\{\hat{H},\Upsilon \Upsilon^{†}{\}}_{\alpha \beta }-\{\Upsilon \Upsilon^{†},\hat{H}{\}}_{\alpha \beta }\\ & \;+div(\{JA\Upsilon \Upsilon^{†},\hat{H}\}-\{\hat{H},JA\Upsilon \Upsilon^{†}\}+i\hbar^{-1}[JA\cdot \nabla \hat{H},JA\Upsilon \Upsilon^{†}]{)}_{\alpha \beta }\\ & \;+div\left(\{{\hat{H}}_{\alpha \gamma },JA\Upsilon_{\beta }^{*}\}\Upsilon_{\gamma }-\{JA\Upsilon_{\alpha },{\hat{H}}_{\gamma \beta }\}\Upsilon_{\gamma }^{*}\right)\\ & \;+\Upsilon_{\gamma }\{JA\cdot \nabla {\hat{H}}_{\alpha \gamma },\Upsilon_{\beta }^{*}\}-\{\Upsilon_{\alpha },JA\cdot \nabla {\hat{H}}_{\gamma_{\beta }}\}\Upsilon_{\gamma }^{*}\\ & \;+[\hat{H}+JA\cdot \nabla \hat{H}\;,\;\{\Upsilon ,\Upsilon^{†}\}{]}_{\alpha \beta }+{\left\{\hat{H},i\hbar \{\Upsilon ,\Upsilon^{†}\}\right\}}_{\alpha \beta }-{\left\{i\hbar \{\Upsilon ,\Upsilon^{†}\},\hat{H}\right\}}_{\alpha \beta }\\ & \;-i\hbar \{\Upsilon_{\gamma },\{{\hat{H}}_{\alpha \gamma },\Upsilon_{\beta }^{*}\}\}+i\hbar \{\{\Upsilon_{\alpha },{\hat{H}}_{\gamma \beta }\},\Upsilon_{\gamma }^{*}\}. \end{array}$$

Then, expanding the first two divergences in the second line yields equation (3.9).

At this stage, we can verify relations (3.10) explicitly. We begin by proving the first in (3.10), that is, by computing $\int \partial_{t}\hat{D}\;d^{6}z$. This is easily done by using the relation

$$\int \{JA\cdot \nabla {\hat{H}}_{\alpha \gamma },\Upsilon_{\beta }^{*}\}\Upsilon_{\gamma }\;d^{6}z-\int \{\Upsilon_{\alpha },JA\cdot \nabla {\hat{H}}_{\gamma \beta }\}\Upsilon_{\gamma }^{*}\;d^{6}z=-\int [JA\cdot \nabla \hat{H},\{\Upsilon ,\Upsilon^{†}\}{]}_{\alpha \beta }\;d^{6}z,$$

which indeed yields the first in (3.10). Analogously, the second in (3.10) is recovered by computing $Tr\partial_{t}\hat{D}$. The trace of the terms on the right-hand side of (3.9) are obtained as follows:

B 1 $$\begin{array}{rl} Tr[\{\hat{H},\hat{D}\}-\{\hat{D},\hat{H}\}] & =2Tr\{\hat{H},\hat{D}\},\\ Tr[\{JA\Upsilon \Upsilon^{†},\nabla \hat{H}\}-\{\nabla \hat{H},JA\Upsilon \Upsilon^{†}\}] & =-2Tr\{\nabla \hat{H},JA\Upsilon \Upsilon^{†}\}, \end{array}$$

B 2 $$\begin{array}{rl} div(\{{\hat{H}}_{\alpha \gamma },JA\Upsilon_{\alpha }^{*}\}\Upsilon_{\gamma }-\{JA\Upsilon_{\alpha },{\hat{H}}_{\gamma \alpha }\}\Upsilon_{\gamma }^{*}) & =div[Tr\{\hat{H},JA\Upsilon \Upsilon^{†}\}+Tr(\{\hat{H},JA\}\Upsilon \Upsilon^{†})], \end{array}$$

B 3 $$\begin{array}{rl} \Upsilon_{\gamma }\{JA\cdot \nabla {\hat{H}}_{\alpha \gamma },\Upsilon_{\alpha }^{*}\}-\{\Upsilon_{\alpha },JA\cdot \nabla {\hat{H}}_{\gamma \alpha }\}\Upsilon_{\gamma }^{*} & =Tr\{JA\cdot \nabla \hat{H},\Upsilon \Upsilon^{†}\} \end{array}$$

as well as

B 4 $$-i\hbar \{\Upsilon_{\gamma },\{{\hat{H}}_{\alpha \gamma },\Upsilon_{\alpha }^{*}\}\}+i\hbar \{\{\Upsilon_{\alpha },{\hat{H}}_{\gamma \alpha }\},\Upsilon_{\gamma }^{*}\}=-i\hbar Tr\{\hat{H},\{\Upsilon ,\Upsilon^{†}\}\}.$$

The trace of the other terms vanishes. After several computations, one obtains that the sum of (B 1)–(B 4) gives

$$Tr(\{\hat{H},JA\}\cdot \nabla (\Upsilon \Upsilon^{†}))+Tr(\nabla \hat{H}\cdot \{JA,\Upsilon \Upsilon^{†}\})-Tr\{\hat{H},i\hbar \{\Upsilon ,\Upsilon^{†}\}-div(JA\Upsilon \Upsilon^{†})\}.$$

Hence the second equation in (3.10) follows if the following identity holds:

$$Tr(\{\hat{H},JA\}\cdot \nabla (\Upsilon \Upsilon^{†}))+Tr(\nabla \hat{H}\cdot \{JA,\Upsilon \Upsilon^{†}\})=-Tr\{\hat{H},\Upsilon \Upsilon^{†}\},$$

for all $\hat{H}$ and *Υ*. This identity is equivalent to $J(\nabla A-{\left(\nabla A\right)}^{T})J=J$, which follows since $\nabla A-{\left(\nabla A\right)}^{T}=-J$ and *J*^2^ = − 1.
